# To See or Not to See: Do Front of Pack Nutrition Labels Affect Attention to Overall Nutrition Information?

**DOI:** 10.1371/journal.pone.0139732

**Published:** 2015-10-21

**Authors:** Laura Bix, Raghav Prashant Sundar, Nora M. Bello, Chad Peltier, Lorraine J. Weatherspoon, Mark W. Becker

**Affiliations:** 1 School of Packaging, Michigan State University East Lansing, MI, United States of America; 2 Department of Statistics, Kansas State University Manhattan, KS, United States of America; 3 Psychology Cognition and Cognitive Neurosciences Group, Michigan State University East Lansing, MI, United States of America; 4 Department of Food Science and Human Nutrition, Michigan State University East Lansing, MI, United States of America; University of Campinas, BRAZIL

## Abstract

**Background:**

Front of pack (FOP) nutrition labels are concise labels located on the front of food packages that provide truncated nutrition information. These labels are rapidly gaining prominence worldwide, presumably because they attract attention and their simplified formats enable rapid comparisons of nutritional value.

**Methods:**

Eye tracking was conducted as US consumers interacted with *actual packages* with and without FOP labels to (1) assess if the presence of an FOP label increases attention to nutrition information when viewers are not specifically tasked with nutrition-related goals; and (2) study the effect of FOP presence on consumer use of more comprehensive, traditional nutrition information presented in the Nutritional Facts Panel (NFP), a mandatory label for most packaged foods in the US.

**Results:**

Our results indicate that colored FOP labels enhanced the probability that any nutrition information was attended, and resulted in faster detection and longer viewing of nutrition information. However, for cereal packages, these benefits were *at the expense of attention to the more comprehensive NFP*. Our results are consistent with a potential short cut effect of FOP labels, such that if an FOP was present, participants spent *less time attending the more comprehensive NFP*. For crackers, FOP labels increased time spent attending to nutrition information, but we found no evidence that their presence reduced the time spent on the nutrition information in the NFP.

**Conclusions:**

The finding that FOP labels increased attention to overall nutrition information by people who did not have an explicit nutritional goal suggests that these labels may have an advantage in conveying nutrition information to a wide segment of the population. However, for some food types this benefit may come with a short-cut effect; that is, decreased attention to more comprehensive nutrition information. These results have implications for policy and warrant further research into the mechanisms by which FOP labels impact use of nutrition information by consumers for different foods.

## Introduction

Given growing rates of obesity and associated increases in health costs, morbidity and mortality [1–4], numerous governments world-wide have expressed an interest in reducing obesity rates. As part of this effort, Front of Pack (FOP) nutrition labels are becoming prominent internationally [5]. These labels take varied forms, ranging from symbols that provide a global synopsis of a product’s overall healthfulness in summative fashion to those which include explicit details regarding key components commonly associated with health risks when consumed in excess (e.g. saturated fat, fat, sugar and salt). Increasingly, explicit labels are also overlaid with qualitative assessments relating to the specific components highlighted (e.g. a traffic light system, in which red stands for “eat sparingly”, amber for “eat in moderation” and green for “eat up”).

In response to the increased use of FOP labels, and governmental calls for research investigating their effectiveness (Nathan, Lichtenstein, Yaktine, & Wartella, 2011; Office of Foods, Center for Food Safety and Applied Nutrition, Center for Veterinary Medicine, & Office of Regulatory Affairs, 2012; White House Task Force on Childhood Obesity, 2010), there has been a great deal of recent research on FOP labeling.

In this study we fill two critical gaps in the existing literature.

- First, we directly measure how FOP labels impact attention among participants not engaged in a nutrition-related task.
- Second, we investigate how the presence of an FOP impacts attention to existing, more comprehensive, nutrition information (i.e., the Nutrition Facts Panel (NFP) in the US).

To do so, we employ a novel method that allows high-resolution eye tracking while people interact with commercial quality packages.

## Background

**Fig 1:**
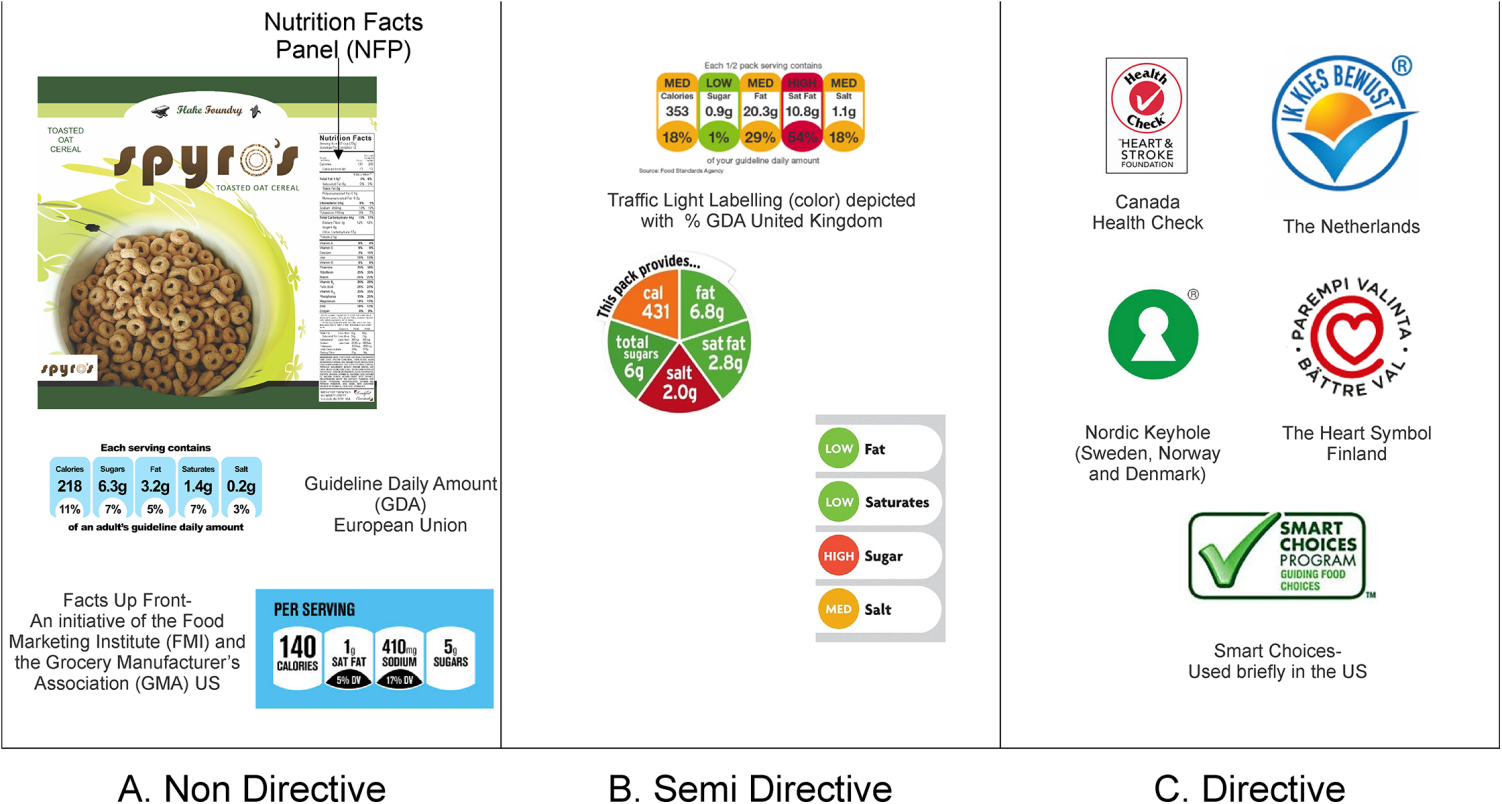
(1A) Examples of Non-Directive labels (1B) Examples of Semi- directive FOPs (1C) Examples of Directive FOPs.

Hodgkins et al. developed a system to classify FOP labeling as: directive, non-directive and semi-directive, and defined “directiveness” as the degree to which the labeling provides guidance about a food’s overall healthfulness [6]. ‘Non-directive’ labels simply offer a listing of nutrients and leave the burden of making the actual healthfulness interpretation to the consumer. Monochrome systems like the %GDA label in the EU and the Facts-up-Front program (also known as Nutrition Keys) [7], as well as the traditional Nutrition Facts Panel (NFP) in the US, are examples of non-directive labeling (Fig 1A). When overlaid with symbols or other qualitative assessments such as color, non-directive labels become ‘semi-directive’, offering a listing of key components while also providing a color-coded interpretation of the healthfulness of each relative to reference amounts (Fig 1B). Finally, simple icons, like the Choices Icon in the Netherlands [8], the Nordic Keyhole-utilized in Norway, Sweden and Denmark [9], and the now defunct Smart Choices label in the US [10] are examples of ‘directive’ labels, those which summarize and directly call out the overall healthfulness of a product in summative fashion (Fig 1C). Regardless of the approach taken (directive, semi-directive or non-directive), the rationale behind FOP labels is that presenting information regarding nutritional aspects of the product in a conspicuous and easy to understand manner will empower people to make healthier choices [11–14].

Most of the research investigating FOP labels focuses on the consumer’s ability to comprehend and use labels of various designs (for review see Vyth et al., 2012). With this focus, many studies have given people nutrition-relevant tasks and evaluated how well they perform the task which requires nutrition labeling, generally during trials which employ varied label designs. For instance, Borg & Westernhoefer (2009) asked people to select the healthy option in a two-alternative forced-choice scheme using packaging designs that included directive, semi directive and non directive methods of labeling. Researchers found that participants performed the selection task better with a color-coded FOP label, a type of semi-directive label. This finding was replicated by Australian researchers, who found that participants were five times more likely to correctly identify a food as healthy when food products were labeled with a traffic light FOP label (i.e. semi-directive) as compared with those which were monochromatic (i.e. non-directive) [15]. In general, it seems that color-coded FOPs, like the multiple traffic light design, are effective when people are asked to identify healthy foods (see Hawley et al 2013 for review).

While this approach is important for identifying labels that are effective *for people who have the goal of recognizing healthy food products*, published research provides little information about a label’s impact on individuals who might not have an explicit nutrition-related goal. From an information-processing prospective, the existing literature focuses on a relatively late process, namely comprehension, by-passing the earlier stage of attentional selection. Bypassing the attentional interaction ignores the fact that one of the reasons to place nutritional information on the front of packs is to make it readily accessible so that it garners more attention [16]. Further, a great deal of research suggests that information must be attended in order to be consciously perceived [17–20]. In short, if a label fails to garner attention, then the cognitive processing of the label will be derailed before comprehension even becomes an issue; that is, if the consumer does not see the information, information processing gets truncated before reaching the point of understanding.

Recognizing this gap in knowledge, recent studies have sought to investigate how different types of nutrition labels impact attention. A number of these studies use visual search tasks to investigate attention to nutrition labels. Many employ the reaction time required to find a nutrition-relevant search target as the dependent variable [21, 22]. Others have tracked eye movements while participants were given explicit, nutrition-relevant goals like identifying whether a product had low or high sodium content, or making a health judgment about a product [23–28]. These visual search paradigms generally support the claim that semi-directive (specifically, color–coded) FOP labels are effective at attracting attention. However, in all these visual search paradigms the participants were given nutrition-relevant tasks, predisposing them to attend the relevant information. So, much like research on comprehension, these studies are unable to speak to the ability of labels to garner attention from people who do not have an explicit nutritional goal.

Other researchers have eye tracked participants while explicitly manipulating participants’ goals in order to compare those with a nutrition-related goal to others with taste or purchase-related motivations [21, 22, 29–32]. In general, these studies show that participants with a nutrition related goal spend more time attending to nutritional information and that formats akin to the Traffic Light FOP label garner attention particularly well among that population [21, 22]. What is missing from these studies is an assessment of whether FOP *design* impacts the attention of those participants that do not have an explicit goal related to nutrition. In other words, the results do not speak to the question of *how effective* FOPs are among the general population. It is still unclear which designs best garner attention *among participants without a nutrition relevant task*.

The distinction between participants with and without a nutrition-related goal is an important one practically and theoretically. From the practical standpoint, a label that garners attention, even among participants who are not explicitly seeking nutritional information, is likely to impact a greater segment of the population. From a theoretical standpoint, the two scenarios (with and without a nutritional goal) tap different attentional systems. Two systems are generally regarded as governing attention: a bottom-up system and a top-down system [33]. These systems are associated with distinct neural pathways, and have different time courses. When information search is goal-driven, attention is driven in a top-down fashion toward goal-relevant stimuli; whereas in the absence of goals, attention wanders to the most visually salient stimuli [33]. As a result, it is unclear whether or not a label that works well under volitional (top-down conditions) will also perform well under involuntary bottom-up conditions.

Evidence from a single eye tracking study that investigated attention to packages with varied FOP labeling strategies among participants who were not given a nutritional goal is available [34]. A mobile eye tracker was used to track people’s gaze during a mock shopping task by recording the duration of time that each person gazed *at the entire package* as well as the number of times that their gaze was directed to the same. Presence or absence of a directive FOP (health logo) was crossed with that of a semi-directive FOP (traffic light), forming four possible label treatments. Under the guise of a shopping scenario, participants were asked to make a single selection from each of three categories of food products (ready meals, sweets and fruit juices) while their eye movements were tracked. Researchers reported subjects spent a significantly longer time gazing at packages that contained a traffic light label compared to those that did not. However, the method only allowed the research team to make inference regarding eye movement with regard to the entire package. This raises the possibility that the FOP did attract attention to itself but whether or not the nutrition information was explicitly attended remains unclear.

Interestingly, Koenugstorfer’s team noted that the presence of the semi-directive traffic light label interacted with the appearance of the directive design, a “Health Check Mark” designed to provide an overall evaluation of the healthfulness of the product, a directive design. When the Health Check appeared alone, people spent very little time viewing packages. But when the package displayed both a Health Check and the traffic light, people looked at the packages for significantly longer. This finding suggests that the traffic light was attended, altering the impact of the Health Check. The finding that the traffic light label may have altered the use of other nutritional information highlights a second gap in our knowledge. Specifically, that there is little data investigating how the presence of an FOP may impact attention to the more comprehensive information presented in the Nutritional Facts Panel (NFP).

In our study, we employ a high-resolution eye tracking method that enabled us to isolate fixations on specific sections of the package to more precisely detect the attentional patterns when people without explicit, nutrition-related goals interacted with actual packages with and without FOP labels. This is important, because it has been suggested that the FOP could act either as a prime or shortcut for the more comprehensive, required nutritional information contained within the NFP.

Determining whether the FOP primes or substitutes more complete nutrition information is an important area of inquiry for two reasons. Koenugstorfer’s work [34] supports the idea that the presence of different types of nutrition labels can influence the attention devoted to others. Additionally, it has been argued that an over-reliance on the limited nutrition information in an FOP may reduce a consumers’ ability to decide what is most appropriate for him/her based on individual needs [35, 36] (i.e. consumers should access the comprehensive information to make truly informed decisions regarding appropriate dietary choices).

Our experiment allowed us to objectively investigate what portions of each package participants (not tasked with a nutrition-related task) viewed.

## Objectives

Using an Applied Science Laboratories 501 bright pupil, head mounted eye tracker (ASL Boston, MA), we tracked the gaze position of 55 participants as they interacted with novel cereal and cracker packages created for this research in order to:

1. Determine whether the presence of an FOP increased attention to nutrition information in participants that were not specifically charged with a task that required nutrition information.
2. Study the interplay between FOP labels and existing, comprehensive nutrition information (i.e. the NFP) to address whether the FOP is used in lieu of the NFP or primes nutritional information, making people more likely to use the NFP.

## Materials and Methods

### Participants

This study was approved under IRB 10–459 by the Social Science/Behavioral and Education Institutional Review Board (SIRB) at Michigan State University. Participant eligibility required: 18 years or older, not legally blind, and hard contact non-user (can interfere with the eye tracking device). Written consent was provided by all subjects and data was recorded by subject number.

To obtain a diverse sample that represented at-risk participants, we recruited adult participants from Michigan State University (MSU) campus list serves and extension programs housed at the Ingham County Health Department. The programs that helped us recruit participants from at-risk communities included: the Women Infants and Children (WIC) program, the Supplemental Nutrition Assistance Program (SNAP) and the Expanded Food and Nutrition Education Program (EFNEP) with the assistance of MSU Extension (MSUE). These are government supported programs that assist people with limited resources in acquiring the: food, knowledge, skills, attitudes and behaviors necessary for nutritionally sound diets

We recruited a total of 74 participants, 12 of whom were not included in the study due to technical difficulties with the instrument calibration process, and hence their eye movements could not be tracked. Data collected from an additional seven subjects were excluded from analysis due to poor quality of the captured track (e.g. excessive eye blinks, movement of the head, etc.). As such, data from 55 (31 Female) participants were used for analysis. Participants ranged in age from 18 to 72 (M = 36.6, SD = 14.3). Table 1 presents the participant characteristics in terms of ethnicity, household income, education level, and weight status. For the purpose of statistical analyses, educational level was grouped into two categories, those that had completed a high school degree (or more) and those that had not. Similarly, income level was grouped into two categories, those who reported household annual incomes of less than $20,000 and those who earned more.

**Table 1:** Participant Characteristics.

Characteristic	Number of	Percent
Ethnicity		
White/Caucasian	23	41.8%
Black/African American	15	27.3%
Hispanic	2	3.6%
Asian	14	25.5%
Did not report	1	1.8%
Household Income		
<$20,000	32	58.2%
$20,000-$49,999	15	27.3%
$50,000–74,999	0	0%
$75,000–99,999	1	1.8%
<$100,000	4	7.3%
Did not report	3	5.5%
Education		
Less than High School	8	14.5%
High School Graduate	23	41.8%
Bachelor’s Degree	5	9.1%
Graduate Degree	19	34.5%
Body Mass Index Status		
Under weight(BMI <18.5%)	0	0%
Healthy Weight (BMI 18.5–24.9)	18	32.7%
Over weight (BMI 25–29.9)	16	29.1%
Obese (BMI > 29.9)	18	32.7%
Did not participate	3	5.5%
Primary Household shopper		
Yes	45	81.8%
No	10	18.2%
Children in Household		
Yes	17	30.9%
No	38	69.1%

### Eye tracking

**Fig 2:**
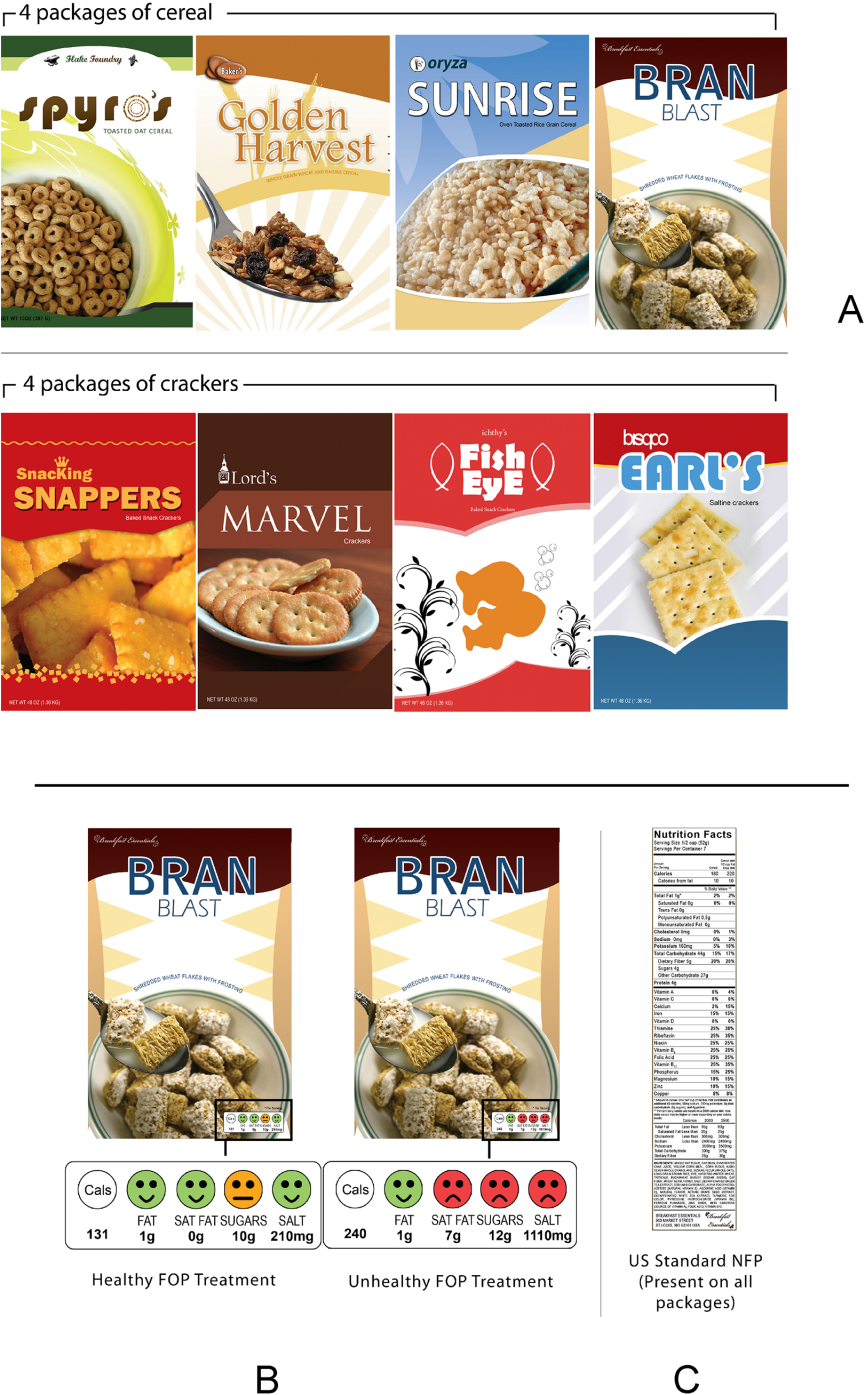
(2A) Principle display panels (PDP for the four brands of cereal (top row) and four brands of crackers (middle row) that were created (in the form of packages) for the experiment. (2B) PDP of a single brand of cereal depicted at high and low levels of health which include the corresponding traffic light labels. (2C) Illustration of the standard Nutrition Facts Panel (NFP) that appears on the panel immediately to the right of the PDP on cereal boxes.

Participants were seated at a desk that was set up either in a university lab or a room in the Ingham County Department of Human Services Building. The desk had a fixture comprised of a mounted sheet of clear plastic (used as a calibrated viewing plane) and a padded chin- rest that was used to maintain each participant’s head position. Using a 9-point floating technique, the eye tracker was calibrated to the plane for each individual. This calibration technique is described thoroughly in our previous publications [37, 38], and enables us to accurately track the gaze of participants while they interacted with actual food packages; specifically we examined behavior while subjects interacted with brands of cereal and crackers that we created for the study (Fig 2A).

Once calibrated, the following instructions were read to participants,

*“We are interested in how people perceive box labels*. *We’re going to show you packages for some new crackers and cereal*. *Please look at these packages as if you were thinking of buying them*. *You will see 8 packages*. *Please continue to look at each until I tell you to stop*. *You may look at any side of the package*, *but please press the package against the plastic pane while viewing it so that we can accurately track your eyes*.*”*

Published times that consumers spend viewing products when making a purchased decision are varied; ranging on average from 12.2 seconds [39] collected in a store environment to 25 seconds measured as part of an eye tracking study [40]; these times mark a range consistent with gaze durations reported more recently by Koenigstorfer et al. [34]. As such, our participants viewed each package for a period of 20 seconds (as measured with a stop watch) while we tracked their eye movements. Order of package presentation was randomized across participants to mitigate any potential order effects.

#### Stimuli

Four novel brands of crackers and four novel brands of cereal were created for use in the study (Fig 2A). For each brand of cereal and of crackers, we manufactured four boxes corresponding to factorial combinations of 2 levels of FOP (present/absent) X 2 levels of health (healthy/unhealthy), thus yielding a total of (2 FOP levels x 2 health levels x 2 products x 4 brands per product =) 32 boxes. Any given participant saw only eight boxes consisting of the 8 product-brand combinations. In turn, any given participant saw each of the 4 labeling conditions (2 FOP levels x 2 health level) on one box of crackers and on one box of cereal. The combination of 4 labeling conditions and 8 product-brand combinations was counterbalanced across participants to prevent confounding between labeling treatments and packages.

All packages were created using ESKO ArtiosCAD®. Graphics were designed using Adobe Photoshop CS3® and printed on a HP Designjet 4520ps large format plotter on Economy Photo Satin paper supplied by Graphix Universal. These printed graphics were pasted on 16 pt coated Solid Bleached Sulfate (SBS) using a Xyron® 4400 Laminator. Blanks were then cut using a Flatbed Kongsberg® 1930 Sample cutting table and pasted using Grainger® thermal glue. This process resulted in high-resolution, commercial quality, real packages, that served as the experimental stimuli.

Nutrients/ingredients of interest were categorized into red/amber/green (with appropriate facial icon) based on the high/medium/low Traffic Light Label guidelines released by the Food Standards Agency based on a 30 gram serving [41]. “Healthy” treatments had three green/smiling icons out of four components featured on the FOP label, namely sugar, salt, fat and saturated fat (Fig 2B). In turn, “unhealthy” treatments had three red/frowning icons out of the 4 nutrients presented on the FOP label (Fig 2B). The values for both healthy and unhealthy versions were within the range of commercially available products. All remaining components not included in the FOP label were held constant, as were the lists of ingredients. All packages contained the standard NFP on their right side (Fig 2C). The information in the NFP always matched that in the FOP with only the components that appeared within the FOP label changing across examples of the same brand.

An additional feature of our FOP labels was the use of schematic facial icons, smiling/straight/frowning faces used as a redundant cue to the color-coded FOP [42]. A large body of evidence suggests that a face, even an ‘iconic’ face like the one used in our FOPs, incites attention [43] [44]. Cognitive research further indicates that facial expressions of emotion are rapidly evaluated [45–47].

#### Statistical Analyses

Eye tracking videos showed the participants view with the location of gaze superimposed over the video footage that was collected as participants interacted with the various packages. GazeTracker® software was used to define two areas of interest (AOI), namely the NFP and the FOP, on each frame of the eye tracking video. With the AOIs defined, the GazeTracker software coded the time to first fixate the AOI, the number of gazes at the AOI, and the total gaze duration within the AOI.

Generalized linear mixed models were fitted to each response variable. A normal distribution was assumed and responses were log or square-root transformed, as appropriate for each variable, to meet model assumptions. For all models, the linear predictor included fixed effects for label conditions (i.e. FOP label present vs. absent), health status (“healthy” vs “unhealthy”) and product type (crackers vs. breakfast cereal—Fig 2B), as well as 2- and 3-way interactions. The linear predictor also considered the random effect of subject fitted as a blocking factor, as well as its cross product with the main effect to properly recognize their level of replication (i.e. experimental units). Random effects of brand nested within product were considered but removed from the final model as the corresponding variance components converged to zero. Demographic variables, including BMI category, age, gender, income level, education level, color blindness, visual acuity, and presence of children in the household were considered as potential explanatory covariates and were included in the modeling process whenever they were found to improved model fit.

Degrees of freedom were estimated and standard errors were adjusted using Kenward-Roger’s procedure. Relevant pairwise comparisons were performed using either a Tukey-Kramer or a Bonferroni adjustment, as appropriate in each case, to prevent inflation of Type I error. Following inference, estimated means of interest were backtransformed and are presented in the original scale, along with corresponding 95% confidence intervals, indicated by M_est_ and CI_95%_, respectively. All models were fitted using the GLIMMIX procedure of SAS (Version 9.2, SAS Institute, Cary, NC).

## Results

### Does the presence of an FOP result in greater attention to nutrition information?

One of our main objectives was to determine whether the presence of an FOP increased attention to nutrition information in participants that were not specifically charged with a task that required it (Objective 1). To explore this issue we investigated

- whether the presence of an FOP increased the probability of people attending *any* nutritional information (either the FOP or the NFP)
- whether FOP labels increased the speed with which nutritional information (either the FOP or the NFP) was attended, and
- the overall time spent attending to nutritional information (the FOP and the NFP).

To do so we performed analyses comparing attention to *any* nutrition information when an FOP label was present or absent from the package.

#### Probability of viewing nutrition information of any type

The probability that nutrition information of *any kind* was noticed was significantly greater (P = 0.0013) for packages with colored FOP labels (M_est_ = 99.6, CI_95%_[97.5,99.9]) compared with NFP-only packages (M_est_ = 90.9%, CI_95%_[81.5, 95.8])). This was the case regardless of whether participants looked at packages of crackers or breakfast cereal at healthy or unhealthy levels of content.

To compliment this finding, we conducted an additional evaluation and considered whether the FOP was more effective than the NFP at attracting attention. For this purpose, we compared the probability of fixation of the FOP vs that of the NFP in the subset of stimuli in which both labels were present. In these conditions there was no evidence that participants were more or less likely to fixate the FOP than the NFP (M_est_ = 100% vs 99.9% respectively, CI_95%_ [99.3, 100] for both, P = 0.979). This may be partially explained by the fact that both labels were fixated at near ceiling levels (i.e. 100%), likely because participants viewed each package for a total of 20 seconds, which constitutes a relatively long time for decision making at point of purchase. Studies report average times for decision making ranging from 12 seconds [39] to 25 seconds [40].

#### Time to first fixation of nutrition information of any type

Analysis of the time to first fixation also supports the use of FOPs. Overall, nutrition information was noticed over 3 times quicker (P<0.0001) on packages that displayed FOP labels (M_est_ = 2.4s, CI_95%_[2.0,2.9]) compared to packages displaying only the current US standard for nutrition information, namely the NFP (M_est_ = 7.9s, CI_95%_[7.0, 8.8]). This effect was apparent for both products (i.e. crackers and cereals), regardless of the health level of the food content.

To further compliment this finding, we considered how rapidly FOPs and NFP attracted attention. For this purpose, we compared the time to first fixate the FOP vs time to first fixate the NFP in the subset of stimuli in which both labels were present. This analysis showed a clear FOP advantage, whereby the FOP was first fixated much earlier in viewing than the NFP (M_est_ = 2.25, CI95% [1.80, 2.75] seconds vs. M_est_ = 9.72, CI_95%_ [8.84, 10.65] seconds, respectively; p = 0.0001).

**Fig 3:**
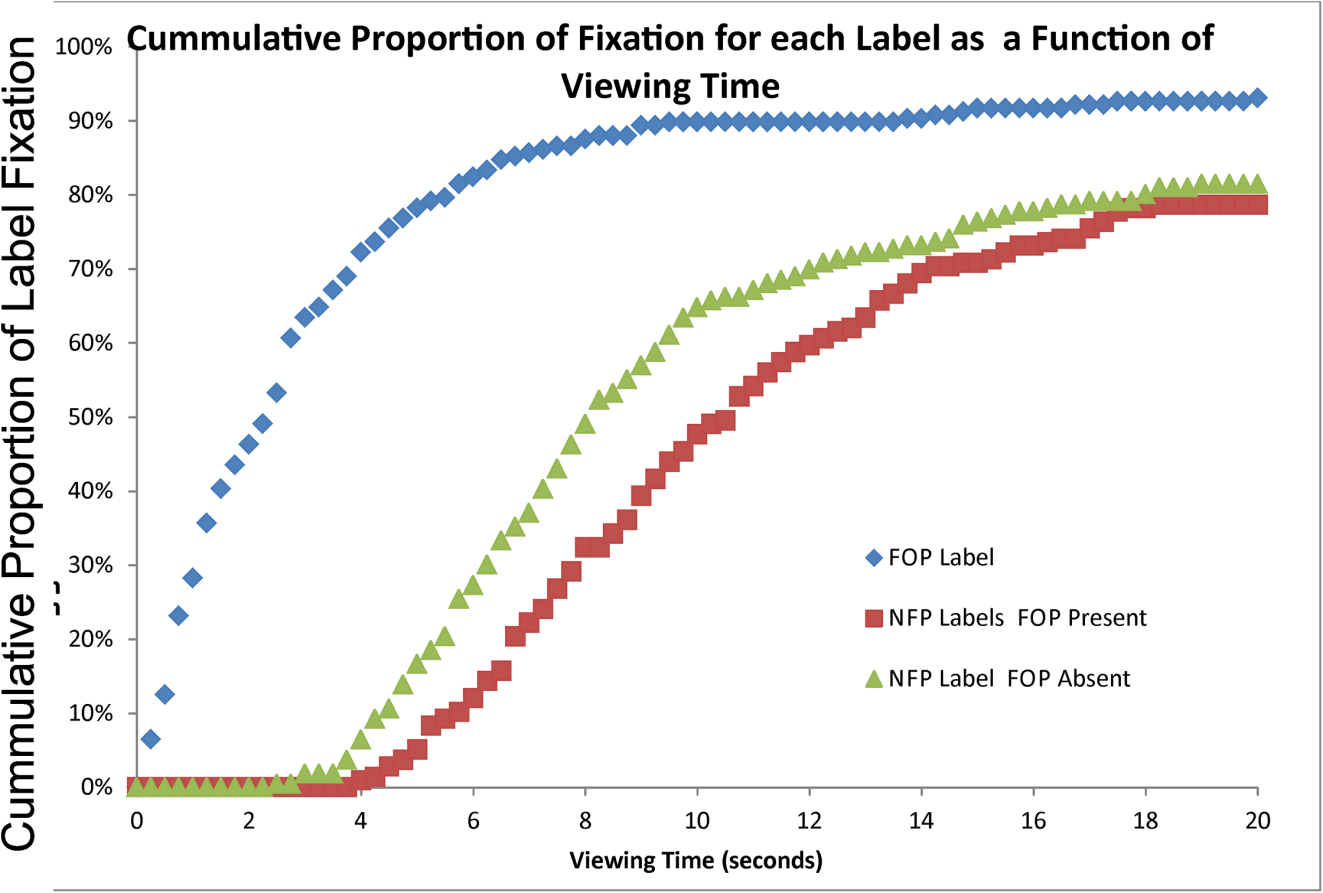
Plots the percentage of each type of nutritional label that has been fixated as a function of viewing time. Data were collapsed across participants so the percentage was based on the number of labels fixated out of the 220 total labels per label type (4 labels x 55 participants)

To further visualize the dynamics with which FOP and NFP labels attracted attention, Fig 3 presents the cumulative proportion of all FOP and NFP labels that were fixated as a function of viewing time during a trial. Studies indicate time to purchase decision to be relatively short [39, 40], thus suggesting that the creation of nutrition information that captures attention quickly is imperative. Note that by five seconds into viewing time, about 80% of the FOPs had been attended, whereas fewer than 20% of the NFPs had been attended by this point, regardless of whether the stimuli contained an FOP or not.

#### Total viewing time of nutrition information of any type

In addition to faster times to fixate nutrition information, participants spent more time viewing nutrition information of any kind when the package had an FOP label as compared to those without (M_est_ = 6.1, CI_95%_[5.2,7.1] seconds vs. (M_est_ = 5.2, CI_95%_ [4.4, 6.2] seconds, respectively) (P = 0.0032). After accounting for labeling conditions, product types (cereal vs. crackers) also differed on the total time that people spent viewing nutrition information (P = 0.0070), whereby more time was spent on the cereal products ((M_est_ = 6.10, CI_95%_ [5.15,7.13] seconds) when compared with the crackers ((M_est_ = 5.25, CI_95%_ [4.37, 6.20 seconds]). Further, the health level of both cereal and cracker products also differed in total viewing time of the nutrition information (P = 0.0096) that was presented. Specifically, unhealthy products generated significantly longer total time spent on nutrition information ((M_est_ = 6.0, CI_95%_ [5.07,7.00] seconds) than those produced at healthy levels ((M_est_ = 5.34, CI_95%_ [4.47,6.29] seconds]).

### Does the FOP act as a prime for, or eclipse more comprehensive nutrition information?

Our second objective was to study the interplay between FOP labels and existing, comprehensive nutrition information (i.e. the NFP) to address whether the FOP is used in lieu of the NFP or primes nutritional information, making people more likely to use the NFP. To explore this issue we investigated

- the probability of viewing the NFP as a function of the FOP presence
- the time to first fixate the NFP as a function of FOP presence
- the total time spent viewing the NFP as a function of FOP presence
- the number of visual hits to the NFP as a function of FOP presence

#### Probability of viewing the NFP as a function of FOP presence

There was no evidence for any effect of presence of FOP labels on the probability of detecting the NFP (P = 0.59), neither any evidence for differences between product types or health levels (P = 0.60 and 0.68, respectively). The estimated probabilities of fixating the NFP ranged approximately from 85 to 95% across labeling conditions and products. As mentioned in a previous section, such high fixation rate may be partially explained by the relatively large amount of time that participants were allowed to view the packages (i.e. 20 seconds).

#### Time to first fixation on the NFP as a function of FOP presence

We detected evidence for a main effect of FOP presence on the time to first fixate the NFP (P<0.0001), such that fixation of the NFP was delayed on packages that had an FOP label relative to those that did not (M_est_ = 9.27, CI_95%_ [8.43,10.2] vs. M_est_ = 7.49, CI_95%_ [6.81, 8.23] seconds, respectively). None of the interaction terms were significant (all P >0.16); that is, NFP fixation was delayed in the presence of an FOP label for both types of products regardless of health level.

#### Total time spent on the NFP as a function of FOP presence

**Fig 4:**
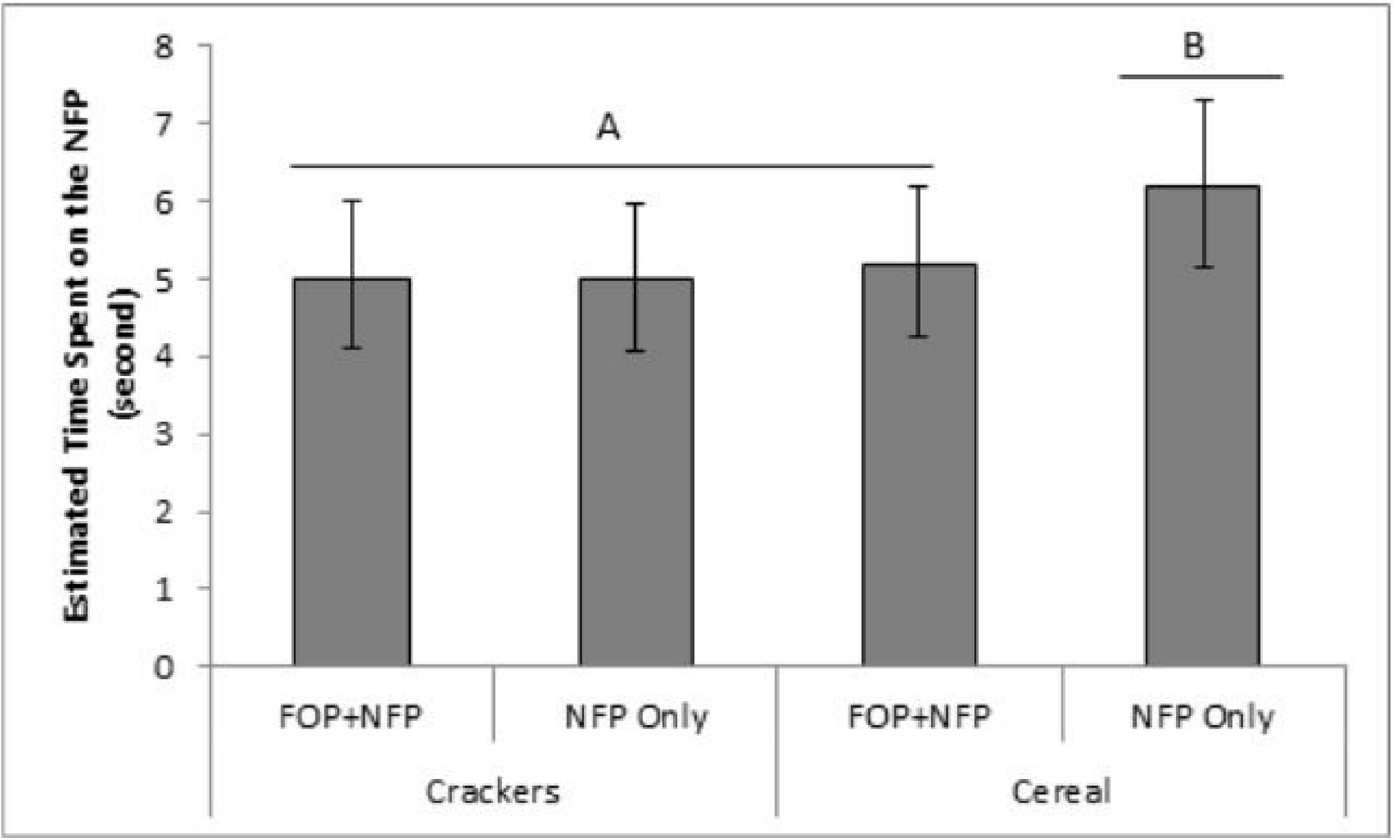
Estimated mean total eye-gaze time spent on the Nutrition Facts Panel for cereal and cracker packages that did and did not include an FOP label. A 2-way interaction was apparent between product type and whether an FOP label was present or not, P<0.05.)

When we examined the total time spent viewing the *NFP only*, we found evidence for a 2-way interaction between product type and whether FOP labels were present or not (P = 0.0486). For packages of breakfast cereal, people viewed the NFP for a shorter time when the FOP was present as compared to absent (M_est_ = 6.2, CI_95%_ [5.2,7.3] seconds vs. M_est_ = 5.2, CI_95%_ [4.2, 6.2] seconds; P = 0.0085). In turn, there was no evidence for any differences in time spent viewing the NFP when cracker packages with or without FOP labels were compared (M_est_ = 5.0, CI_95%_ [4.1,6.0] seconds vs M_est_ = 5.0, CI_95%_ [4.1, 6.0] seconds, respectively; P = 0.95). Follow-up comparisons suggest that the time spent viewing the NFP for cereal packages that did not have an FOP was significantly longer than for other treatments (all p<0.05) (Fig 4).

A main effect of the product’s healthfulness was also evident on the total time spent viewing the NFP (P = 0.0053), with unhealthy products garnering more time on the NFP than products presented at a healthy level (M_est_ = 5.7, CI_95%_ [4.78, 6.64] seconds vs M_est_ = 4.97, CI_95%_ [4.14, 5.87] seconds, respectively).

#### Number of visual hits to the NFP

**Fig 5:**
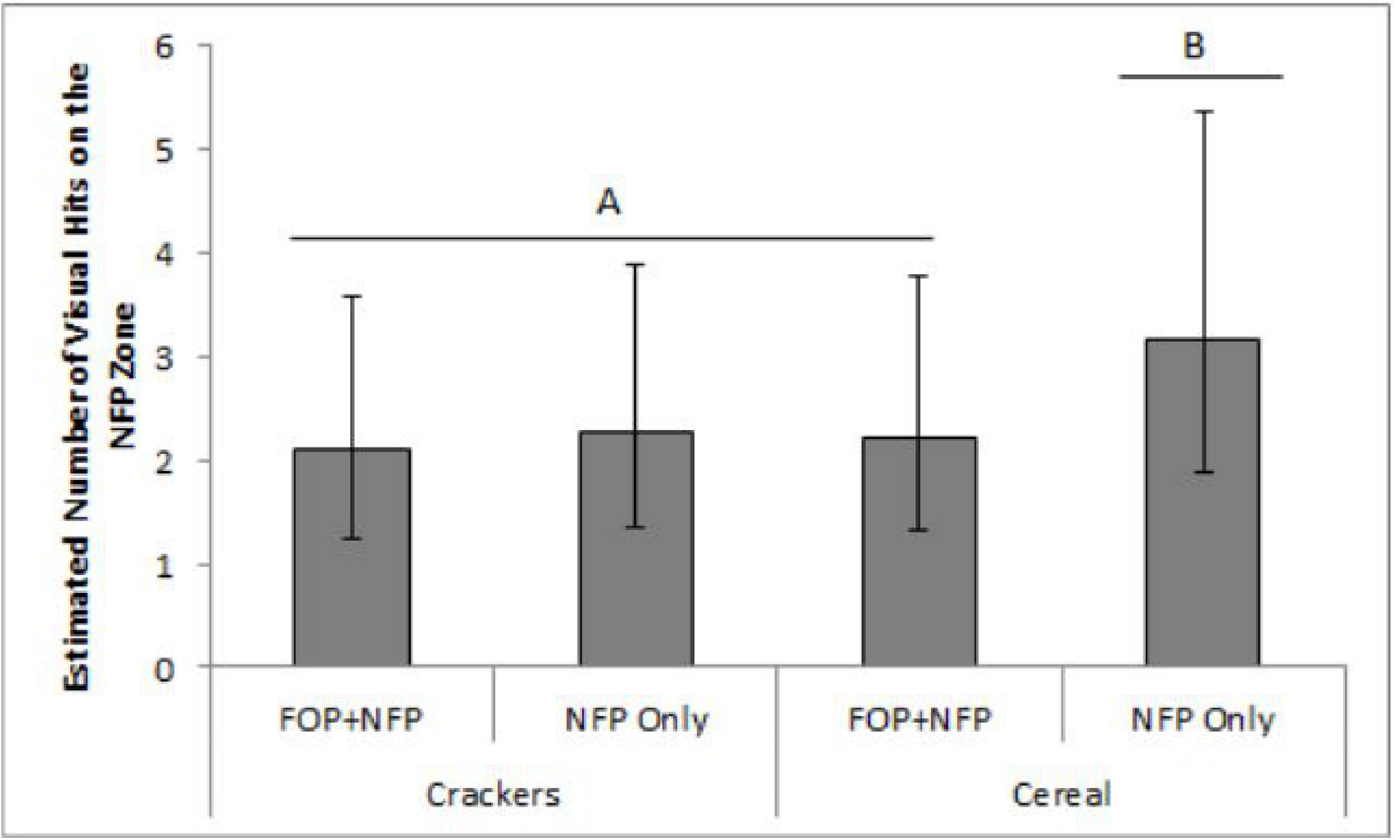
Estimated number of visual hits on the Nutrition Facts Panel for cereal and cracker packages that did and did not include an FOP label. A 2-way interaction was apparent between product type and whether an FOP label was present or not. ^a,b^ P<0.05

A similar pattern was apparent when we considered the number of visual hits to the NFP as the response of interest. A 2-way interaction between product type and the FOP presence was apparent (p = 0.038). Specifically, the number of times a participant’s eyes returned to the NFP depended on whether the FOP label was present or not in cereal packages, but not in cracker packages. Cereal packages had a significantly fewer number of visual hits to the NFP when an FOP label was present relative to when it was not (M_est_ = 2.2, CI_95%_ [1.2, 3.8] hits vs. M_est_ = 3.2, CI_95%_ [1.9, 5.4] hits, respectively; p = 0.0002). In turn, there was no evidence that the presence of an FOP influenced the number of hits on the NFP for cracker packages (M_est_ = 2.1, CI_95%_ [1.2, 3.6] hits when the FOP was present vs M_est_ = 2.3, CI_95%_ [1.3, 3.9] hits in when it was not (p = 0.4) (Fig 5). Follow-up comparison suggests that there were significantly more gazes at the NFP label in the NFP-only cereal condition, that is when cereal packages did not have an FOP label, than in any other product-label conditions (all p<0.02) but that no significant differences were apparent amongst the other three conditions (all P>0.84) (Fig 5).

## Discussion

### Does an FOP result in greater attention to nutritional information?

Our results support the idea that attention to nutritional information on a package takes place more effectively and efficiently in the presence of a colored FOP label. More specifically, FOPs were fixated much earlier in viewing than NFPs. Within the first 5 seconds of viewing, about 80% of FOPs were fixated whereas fewer than 20% of NFPs were by this point in viewing time. Additionally, people spent significantly more time fixating nutrition information (either the FOP or NFP) when a colored FOP was present.

While these findings are broadly consistent with prior work that suggests that colored FOP labels increase attention to nutrition information [48], our data extend previous work in two important ways. First, the effect of FOP was apparent even when people who were not given a nutrition-relevant goal; as such, FOP labels seem likely to engage attention broadly. Second, we directly compared attention to the FOP label relative to the NFP on a given package and found that the FOP was better at attracting attention than the NFP. This type of direct comparison of an FOP to standard nutrition information is important in that it can be used in policy discussions regarding the creation and implementation of new labeling standards.

Interestingly, we also found that participants seemed to spend more time viewing nutrition information when the label indicated an unhealthy food product. This is encouraging as it suggests that people are sensitive to differences in the FOP label content. In addition, the finding that people spend more time attending to the labels depicting unhealthy foods is consistent with prior work that suggests an attentional bias towards and slower disengagement from negative or threatening stimuli [49–52].

The type of food product also impacted overall time spent fixating on nutrition information, whereby people spent more time viewing nutrition information for cereals than for crackers. As a possible explanation of why the nutrition information of cereals was viewed for a longer time than crackers, we speculate that cereals might have been perceived as a meal while crackers might have been perceived as a snack. If so, this would suggest that people are more motivated to view nutrition information when the food is perceived as comprising a greater portion of one’s diet. Consistent with this interpretation, others have found that people spend significantly more time viewing the nutritional information of meals than snacks, in a mock purchasing scenario. [53] Alternatively, one could also explain this result in noting that products that widely range in nutrition value may induce different behaviors than those with more predictable nutrition contents. Under this explanation, people might be more interested in the nutrition information of cereals because they vary greatly, from nutrient dense to nutrient poor alternatives. In contrast, crackers may be considered a more standardized product, in which case nutrition information may be considered less important to assess.

Although not assessed, the presence of a diet-related health problem such as diabetes may also impact the types of foods that are more likely to receive more attention. This is not beyond the realm of possibility, as prior work has found that attention to nutrition information is impacted by factors specific to the viewer. For instance, health motivation (high motivation vs low motivation [54]) and goals (health vs preference or taste [48, 55]) can impact the time that people spend viewing nutrition information. It has also been suggested that the use of front of pack labels impacts food choice for sustained periods [56].

Although our data cannot distinguish between these and other possible alternative explanations, they support the fact that people seem to modify their attention to nutrition information as a function of food product. Additional research investigating how motivations and beliefs about potential nutrition variability in foods is needed in order to optimize labeling strategies on a product-specific basis.

### Does the FOP replace or prime the NFP?

We also studied the question of how the presence of an FOP label impacts consumer behavior with regard to the NFP. This is important, because the presence of FOP labels, which simplify nutrition information substantially, may be used as a short-cut, leading people to ignore the more comprehensive information that is presented elsewhere on the package. In the US, this comprehensive (required) labeling takes the form of the Nutrition Facts Panel (NFP), located on the panel directly to the right of the Principle Display Panel (PDP) (Figs 1A and 2C). Given that a healthy dietary choice for an individual is based on a confluence of factors (e.g., age, sex, activity level, health status, cultural values, and serving size [57]), amongst others, over-reliance on the limited nutrition information in an FOP may reduce a consumers’ ability to make appropriate choices based on their specific, personal dietary requirements [35, 36].

These limitations have led some to suggest that health and nutrition claims should be eliminated entirely from package fronts, and that, instead, efforts should be made to improve existing nutrition labeling [58]. The goal of this approach would be to provide comprehensive nutrition information, similar to that in the currently regulated NFP, but to do so in a format that makes the information more understandable and readily available to the consumer. In direct contrast, others claim that positioning nutrition information on the front of packages is needed. It has been suggested that people with low motivation for healthful choices pay more attention to information on the front of the package than to other panels of the package [59]; and that those with health motivations will use information from the FOP label as a catalyst to access the more comprehensive NFP [54].

We found no evidence that the FOP primes attention to the NFP. On the contrary, our results suggested that the presence of the FOP label on a cereal box lowered the amount of attention to the NFP. Specifically, the NFP was fixated later in the viewing period and for a shorter period of time. This pattern is consistent with the idea that the FOP label replaces the NFP and serves as a short-cut to nutritional information. However, it is important to note that this replacement effect was product-specific as it was only evident with cereals. In fact, the amount of time spent viewing the cereal NFP was relatively high when the NFP provided the only means to evaluate the nutritional value of the cereal (Fig 4).

In short, these data suggest that the simultaneous presence of the FOP on *the cereal package* was used in lieu of the more comprehensive information in the NFP (i.e the short cut effect). As a possible interpretation of this pattern, we speculate that people are more motivated to evaluate the nutrition information for cereals than crackers (see discussion related to total time spent on nutrition information in cereal packages vs crackers). As a result, the amount of time spent viewing the NFP for cereals is relatively high when the NFP provides the only means to evaluate the nutritional value of the cereal. If, however, the FOP is present, it is detected early in the viewing and participants fulfill their need for nutrition information by viewing the FOP. Having addressed that need, they are no longer motivated by nutrition information once they reach the NFP. In short, these data suggest that the simultaneous presence of the FOP on the cereal package was used in lieu of the more comprehensive information in the NFP (i.e the short cut effect).

It is worth noting that our data are different from the one other study that investigated relative attention to the FOP vs. NFP. Turner et al. (2014) eye tracked participants as they viewed packages on a computer screen [54]. Participants were shown the front panel of real products that had a single FOP alongside the product’s NFP (i.e. a flattened view) and were asked to perform a mock-shopping task for another person. There were three shopping scenarios: no information about the subject they were shopping for, that the person they were shopping for preferred healthy foods or that they were shopping for someone that preferred foods that tasted good. They found that people seeking healthy foods fixated both the NFP and FOP more than those seeking tasty foods, and concluded that the FOPs were not used as short-cuts but, instead, as another source of nutritional information. However, there are substantial differences between our study and that by Turner et al. [54]. Turner et al. presented both the front panel and NFP on the same screen at the same time in a flattened view. This may have artificially increased the amount of attention paid to the NFP. In addition, they gave some participants an explicit goal of seeking healthy products, whereas we did not. Finally, the study by Turner et al. used images of commercial packages from the US market. Given that FOPs are not regulated in the US, these are likely to have been FOP systems developed by the product manufacturers. These systems are variable, and can be perceived as unreliable [60], both of which may decrease reliance on the FOP. Further, participants familiarity, or lack thereof with each of the brands may have altered their interactions with the nutrition information.

Regardless, our data show that, in specific instances, the FOP has the potential to serve as a shortcut for the more complete information on the NFP. As such, more research specific to situations where this type of short-cut occurs is warranted.

Overall, the pattern of results we present suggest that the presence of an FOP increases attention to any nutritional information, but that the FOP may, at least for some food products, reduce attention to the NFP.

## Conclusions

Our work strongly supports the idea that FOP labels are effective at garnering attention to nutrition information. The added presence of color-coded FOP labels on food packages attracted attention to nutrition information more rapidly and increased the total time that people spent attending to any nutrition information. However, we also found that FOP labels can be used, under certain situations, as a short-cut, thereby decreasing people’s attention to the more comprehensive information found in the NFP. For the scenarios considered in this study, we found no evidence that the presence of FOP labels primed attention to the comprehensive nutrition information on the NFP. These findings have practical implications for policy. First, our finding that FOP labels can, under some circumstances, reduce attention to comprehensive information in the NFP, suggests that any attempt to standardize FOP labels should ensure that that the most important information for making healthy choices appear in the FOP label. Conversely, this “short-cut” finding suggests that manufacturers should not be allowed to selectively report nutrition information on the front-of-pack, as it has the potential to mislead consumers. However, we believe that the attentional benefits of the FOP outweigh the potential negatives. FOP labels produced a dramatic increase in the speed with which people attended to nutrition information, and increased the overall amount of time spent attending to nutrition information. Moreover, the existing literature suggests that FOP labels may not act as a short-cut when people are explicitly interested in nutrition information [54] and that these labels can catalyze more healthful choices [61] for a sustained period of time [56]. Given the magnitude of the evidence that too few people currently attend to nutrition information [62], raising awareness, even to partial nutrition information, constitutes a step towards fostering informed food choices and potentially impacting overall dietary quality.

In short, FOPs are likely to increase attention to nutrition information among the common shopper who is not explicitly interested in nutrition information. However, the information that appears within the FOP may be particularly influential when evaluating the health of the food, so regulators must carefully consider the type of information that should appear within a standardized FOP.

## Supporting Information

S1 File Raw data—Flat File. (XLSX)

## Abbreviations

AOI: Area of Interest
BMI: Body Mass Index
CDC: US Centers for Disease Control and Prevention
EFNEP: Expanded Food and Nutrition Education Program
FFQ: Food Frequency Questionnaire
FOP: Front of Pack Nutrition Labeling
MSUE: Michigan State University Extension
NFP: US Nutrition Facts Panel
SNAP: Supplemental Nutrition Assistance Program
FDA: US Food and Drug Administration
US: United States
WIC: Women Infants and Children
